# Quality Investigation of Pultruded Carbon Fiber Panels Subjected to Four-Point Flexure via Fiber Optic Sensing

**DOI:** 10.3390/ma18010166

**Published:** 2025-01-03

**Authors:** Zachariah Arwood, Stephen Young, Darren Foster, Dayakar Penumadu

**Affiliations:** Tickle College of Engineering, University of Tennessee, Knoxville, TN 37996, USA; syoung13@vols.utk.edu (S.Y.); drrnfstr@gmail.com (D.F.); dpenumad@utk.edu (D.P.)

**Keywords:** carbon fiber, pultrusion, fiber optic, wind, energy, spar cap

## Abstract

Pultruded carbon fiber-reinforced composites are attractive to the wind energy industry due to the rapid production of highly aligned unidirectional composites with enhanced fiber volume fractions and increased specific strength and stiffness. However, high volume carbon fiber manufacturing remains cost-prohibitive. This study investigates the feasibility of a pultruded low-cost textile carbon fiber-reinforced epoxy composite as a promising material in spar cap production was undertaken based on mechanical response to four-point flexure loading. As spar caps are primarily subjected to flexural loading, large-span four-point flexure was considered, and coupon testing was restricted to tensile modulus and compression strength assessment. High-resolution spatial fiber optic strain sensing was utilized to determine spatial strain distribution during four-point flexure, revealing consistent strain along the length of the part and proved to be an excellent option for process manufacturing quality examination. Additionally, holes with diameters of 2.49 mm, 5.08 mm, and 1.93 mm were drilled through the thickness of full-width parts to determine the feasibility of structural health monitoring of pultruding parts internal to wind blades via fiber optic strain sensing.

## 1. Introduction

Composite wind turbine blades have increased dramatically in size from 9 m blades manufactured in the 1980s producing 100 kW of energy to 60+ meter blades manufactured presently capable of producing 5+ MW of energy [1]. Composites afford key advantages including high stiffness, lightweight, resistance to corrosion, and decreased cost for wind turbine blades. However, as the size of wind turbine blades continues to increase, high stiffness, lightweight construction materials are critical to maintain structural stability and energy efficiency along with a lower levelized cost of energy. Furthermore, the design of the blade must maintain a significant level of stiffness, limiting deflection to avoid potential tower strikes during operation [2,3,4,5]. The spar cap, generally consisting of unidirectional fiber-reinforced composite, is an essential load carrying component of the blade subjected to some of the heaviest loads including both bending and torsion [6,7].

Traditionally, E-glass fiber-reinforced polymers have dominated the market as they are readily infusible and cost-effective. However, glass fibers, having an approximate density of 2.7 g/cm^3^, become cumbersome with large-span blades, whereas the density of carbon fiber is approximately 1.6 g/cm^3^. Additionally, carbon fiber tensile modulus is 2–3 times that of glass fiber, reducing deflection during bending and tensile loading [8]. Unfortunately, commercial carbon fiber has historically been cost-prohibitive, with prices exceeding USD 30/lb, while E-glass fiber costs roughly USD 0.59/lb, but, with the introduction of low cost carbon fibers like Zoltek PX-35, a textile fiber produced from a PAN (polyacrylonitrile) precursor at USD 8/lb, cost obstacles are rapidly vanishing [9,10,11].

Ennis et al. investigated the economic competitiveness of pultruded carbon composites in wind turbine blade manufacturing, and although the material cost increased by 33–55%, the authors reported 17% increase in strength with constant fiber volume fraction and 30–31% reduction in mass, providing more efficient blade design and reduced manufacturing and system costs in a 3 MW wind blade design model [11]. Additionally, Upadhyayula et al. reported an environmental assessment for utilizing pultruded carbon fiber in wind turbine blade manufacturing, focusing on PAN-based virgin fiber-reinforced pultruded composites for spar cap production while shear webs and shells were manufactured with recycled carbon fiber composites. The “hybrid fiber” blades provided increased environmental impact performance, with a 12–89% improvement over their glass fiber-infused counterparts in nine of ten categories, while exhibiting 89% greater marine ecotoxicity from the production of virgin carbon fibers, which releases HCN gas into the atmosphere where it is eventually dispersed into water systems. However, regenerative thermal oxidizers are generally used to capture 97% of these HCN emissions and require negligible energy to run [12].

Pultruded fiber-reinforced composites (P-FRC) are ideal for wind energy applications due to continuous fiber processing, the dimensional stability of pultrusion profiles, consistent mechanical properties, and chemical processing [13,14,15,16,17]. The pultrusion of fiber-reinforced composites is a well-established process utilizing tension during production to produce highly aligned unidirectional composite materials. Briefly, pultrusion processes are capable of rapidly yielding composites with high fiber volume fractions, above 60%, with production rates of approximately 70 cm/min manufacturing lengthy parts in minimal time in contrast to resin transfer systems. To create pultruded composites, large spools of fiber are placed on a creel, and fiber is pulled through a guide into a resin bath. The fiber is then pulled through a heated die to ensure proper geometry, and a tensioner system maintains proper tension to ensure fiber alignment. As the material is pulled through the production line, heat is applied and removed at certain points to reduce cure time, allowing an increased rate of production. As the material exits the pultrusion line, a chopper cuts the parts to specified lengths [18]. Fiber alignment and fiber volume fraction are crucial to ensuring desirable mechanical properties of any composite material. Thus, the ability to obtain optimal mechanical properties while maintaining low production costs makes the pultrusion manufacturing of wind blade sections desirable, specifically for spar caps used to transfer stresses from the blade to the root tube connected to the turbine.

Initiatives to produce a low cost textile-based carbon fiber are being led at the Carbon Fiber Technology Facility (CFTF) in cooperation with Oak Ridge National Laboratory (ORNL), with a goal to produce a material with properties comparable to commercial low-cost carbon fibers which can easily be incorporated into pultruded wind turbine blade spar cap manufacturing [19]. Commercially available Zoltek PX-35 fiber tows contain 50,000 filaments per tow (50 K), while textile fiber tows can exceed 300 K. Large tow bundles coupled with rapid speed of manufacture makes fiber wetting during pultrusion progressively challenging as the part thickness increases. Poor wetting leads to void formation within the part, reducing mechanical properties and giving way to strain localizations responsible for premature failure of the part [20]. Additionally, manufacturing issues like fuzzballs, the localized accumulations of broken fibers that can be pulled through the pultrusion die during manufacturing, create local misalignments of fibers reducing mechanical properties and creating potential failure initiation points [21]. As resistance to flexural loading is the main application issue, spar cap materials are assessed primarily based upon the tensile modulus and compressive strength of the composite part [22]. Thus, there is a need to monitor the spatial strain distribution for P-FRC susceptible to fiber damage and delamination when subjected to flexural mechanical loadings [23]. The development of the pultrusion line in conjunction with varying fiber types and fiber volume fractions required rapid mechanical characterization to capture the properties of the part as produced without coupon extraction. Thus, a large-span four-point loading configuration was adopted utilizing fiber optic strain sensors capable of capturing spatial strain distributions along the length of the part mimicking the practice of structural health monitoring (SHM).

SHM has become increasingly critical to effectively detect damage localization for wind turbine blades [24]. It has been suggested that SHM systems should be able to detect laminate damage areas of 50 mm by 50 mm, adhesive joint cracks 1 m long, and delamination areas 100 mm by 100 mm [25]. Many SHM techniques have been investigated including high- and low-frequency piezoelectric impedance-based and acoustic emission sensors, as well as optical techniques. High-frequency impedance-based sensors provide high-resolution data but require large memory and power consumption, requiring the sensors be placed in close proximity to potential damage accumulation areas to effectively detect the mechanical impedance [26]. Low-impedance sensors often cannot provide sufficient resolution to capture small damage accumulations that quickly lead to catastrophic failure. Acoustic emissions sensing, while promising, is highly dependent upon the geometric features of the blade and the anomaly as well as the physical properties of the constituent materials, requiring a myriad of optimization parameters [25].

Kaewniam et al. provided an excellent summary of state-of-the-art damage detection techniques including traditional strain gages, fiber Bragg grating (FBG) sensors, accelerometers, acoustic emission sensors, and piezoelectric sensors [27]. The authors listed the advantages and disadvantages of each SHM technique. They stated that the major drawbacks of traditional strain gages are as follows: the detection area is based on the size of gage and proximity to the defect, they are not robust for long-term monitoring, and they are sensitive to temperature changes. FBG sensor drawbacks were cost and fragility, whereas the piezoelectric sensor disadvantage was being able to place the sensor close enough to areas where damage would occur. Accelerometers required multiple sensors and an external power source. Similarly, acoustic emission sensing was highly dependent upon proximity to the defect and signal attenuation effects. Du et al. provided another excellent summary of SHM techniques for wind turbine blades, highlighting traditional strain gages, FBG sensors, acoustic emission sensors, and ultrasonic sensing [28]. The authors provided similar insights as Kaewniam et al. for these methods. Kang et al. proposed a piezoelectric sensing method where a laser capable of long distance, large area inspection was used to excite the part allowing detection of a 1 mm diameter hole in a 100 mm by 100 mm inspection area [29]. Although excellent defect detection was reported, the system requires utilizing an Nd-YAG laser, a beam expander, galvanometer scanner, signal conditioner, and decade filter to achieve detection at that resolution.

Fiber optic sensors, consisting generally of fused silica and cladding, offer advantages in monitoring strain for composites parts, due to their small size and flexibility, including wind blade applications [30,31,32,33]. Fiber Bragg grating (FBG) sensors, where strain distributions are measured at a few discrete points governed by etched markings along the fiber optic materials, are widely used for composite structural applications, including the monitoring of the manufacturing of P-FRC [34,35,36,37]. Dvorak et al. had success in utilizing FBG sensors in P-FRC in wind turbine blade static testing, revealing good agreement between FBG sensors and traditional strain gages [37]. For their study, the group embedded the FBG sensors in the spar caps during manufacturing and adhered strain gages locally to provide comparison between the systems. The results from FBG sensors and strain gage measurements were compared to theoretical calculations, revealing excellent correlation.

As an alternative, optical frequency distributed reflectivity (OFDR) has shown promise in civil infrastructure, automotive, and wind energy applications [38,39,40]. High-definition fiber optic sensing (HD-FOS) using OFDR possesses the unique ability to obtain high spatial distribution along the fiber optic sensors, where the measured Rayleigh backscatter (RBS) yields an invariant and repeatable scatter pattern or “fingerprint” based on a function of the position of the fiber optic sensor. HD-FOS using a standard telecommunication grade fiber optic cable affords advantages, including ease of installation (adhering to a surface or embedding) at various stages of composite part manufacturing, immunity to electrical interference, and operating in harsh environments. Additionally, HD-FOS suitable for strain and temperature sensing are lightweight and have small gage lengths offering high spatial resolution [39,41,42,43,44,45].

In a recent civil infrastructure application, a glass fiber-reinforced composite (G-FRC) deck bridge was embedded with an HD-FOS to monitor the strain distribution for a vehicular static loading case study [46,47]. The SHM approach included utilizing two 20 m HD-FOS for the sensor layout, embedded in the top and bottom G-FRC deck panels to monitor the strain distribution of the panel when subjected to static vehicular loading. Furthermore, the case study exhibited a complex tensile and compressive varying strain response spatially for a FRC bridge structure where each HD-FOS consisted of thousands of measurement points along the sensing length [46,47]. Additionally, Souza and Tarpani successfully utilized an embedded OFDR FOS in a multi-directional vacuum-infused G-FRC, reporting strain that correlated well with externally adhered traditional strain gages [48]. The FOS was embedded between layers where the reinforcing fiber changed orientation. The abrupt change in direction of reinforcing fibers allowed void formations along the sensor allowing crack propagation which created light leaks along the FOS reducing the performance of the FOS. However, void formations do not occur when the sensor is embedded in unidirectional G-FRC in the direction of the reinforcement.

In this work, an experimental program was developed highlighting HD-FOS using on-demand sensing capabilities to spatially monitor the tensile and compressive strain behavior of pultruded composite beams subjected to four-point flexural loading as a novel method to monitor the structural health of internal pultruding parts. A new low-cost textile PAN-based carbon fiber developed by the CFTF was compared to commercial low-cost carbon fiber manufactured by Zoltek as reinforcing fibers in the beams. An HD-FOS was used to monitor the tensile and compression spatial strain distribution of the pultruding parts during flexural loadings. The HD-FOS strain response of the panel was compared to strain results of the panel obtained using traditional strain gages. Additionally, a separate panel reinforced with a novel low-cost textile grade fiber was prepared with various diameter holes drilled at four locations, and an HD-FOS was adhered to the surface. The sample was loaded in a four-point flexure configuration, comparing local strains near defects of varying sizes to determine the effectiveness of this technology in structural health monitoring of pultruded internal spar caps of wind blades that otherwise are difficult to assess.

## 2. Materials and Methods

### 2.1. Sensing Principle of High-Definition Fiber Optic Sensors

A commercially available, single channel, optically distributed sensor interrogator (ODiSI-B) unit acquired from Luna Innovations, Inc. (Blacksburg, VA, USA), was used to measure the strain in the tensile and compressive regions of the pultruded composite panels. A high spatial strain resolution as low as 0.65 mm gage pitch interval could be detected with the ODiSI-B interrogator unit. Although traditional strain gages can be used to achieve such a resolution, the spot-wise placement of individual gages forces interpolation between gages, which allows the likelihood of unrecognized strain localizations to increase. The spatial resolution of HD-FOS is achieved through swept wavelength interferometry (SWI) and Rayleigh scattering, where the RBS is measured as a function of the position of the optical fiber. The HD-FOS consists of induced random fluctuations within the fiber core refractive index where the measured RBS spectrum produces a repeatable, static, and unique “fingerprint” to each position along the fiber. A change in strain or temperature is calculated based on the physical location of scattering in the sensing fiber corresponding to a spectral shift in the scattered light, resulting from the applied strain or temperature. A standard relationship of spectral shift, Δu, or frequency shift measurement, Δv, along the sensing fiber is relative to a tared or reference state profile of the fiber under test (FUT) measured in an ambient state and compared to fiber subjected to a change in strain or temperature. According to LUNA, for each fiber segment, j, the reflection spectrum, U, is represented as Uj(v), where v is the optical frequency. When the sensing fiber is subjected to a change in strain or temperature, the segment j undergoes a change in the reflective spectrum, Δu_j_, and optical frequency shift, Δv_j_, and can be expressed as U_j_(v-Δv_j_). A shift in reflection spectrum, Δu_j,_ is calculated by a cross-correlation operation performed on U_j_(v-Δv_j_) and U_j_(v). This spectral shift is analogous to a resonance wavelength, Δλ, of Bragg grating and can be simplified into the following form:

(1) $$\frac{∆\lambda }{\lambda }=\frac{∆v}{v}=K_{\varepsilon }\epsilon +K_{T}T,$$

where λ represents the mean optical wavelength and v is the frequency. The K_ε_ and K_T_ are calibration constants relating to a spectral shift change in strain, ε, and temperature, T, values. Using Equation (1), a change in strain can be represented in the following form in the absence of a temperature change:

(2) $$\varepsilon =-\frac{\bar{\lambda }}{cK_{\varepsilon }}∆v,$$

where $\bar{\lambda }$ represents the center wavelength of the scan and the speed of light is denoted as c. Similarly, following relationship for a temperature change, ΔT, can be simplified from Equation (1) in the absence of a change in strain:

(3) $$∆T=-\frac{\bar{\lambda }}{cK_{T}}∆v.$$

The strain measurement is based on the quality factor, correlating the strength of a measurement compared to the reference spectra, where a strong correlation peak is 1 and a weak correlation peak has value approaching 0. The LUNA software, ODiSI 6, version 2.4.2, has a noise floor of 0.2 to 0.3 for the quality factor. Furthermore, the sensors used in this work are polyimide-coated, fused silica, single-mode fiber optics with an intrinsic strength of approximately 14 GPa and measurable strain values range of ±50,000 με when “proof” tested to 690 MPa. The ODiSI-B interrogator uses a swept range of 40 nm, where HD-FOS, consisting of termination and angled physical contact (APC) connectors, has the capability to measure a strain value range of ±10,000 με. These conservative strain range values indicate that the HD-FOS are suitable for the monitoring of flexural structures subjected to mechanical and temperature loadings [40,43,46,49,50,51,52].

Strain distribution values are based on the measured spectral shift from the ODiSI-B interrogator and can be denoted using the following polynomial relationship:

(4) $$\varepsilon \left(v\right)=C_{1}g+C_{2}g^{2},$$

where v is the optical frequency, ε is the strain (μm/m), and g is the spectral shift.

Equation (4) and Table 1 relate the coefficients C_1_ (με/GHz) and C_2_ (με/GHz^2^) to the strain, ε, and spectral shift, g, where the negative coefficient values indicate an increase in strain [39,53]. In this study, longitudinal strain distribution response was measured using a gage length of 1.3 mm corresponding to gage pitch of 0.65 mm and a data acquisition rate of 23.8 Hz using the ODiSI-B interrogator unit [40].

### 2.2. Pultruded Carbon Fiber Panel Production

Pultruded carbon fiber-reinforced epoxy (Hexion RSL-4597 resin with Epikure CCA-138 curing agent, Hexion, Columbus, OH, USA) panels were manufactured by the CFTF in Oak Ridge, TN, USA, using a low-cost textile carbon fiber, labeled A, or a low-cost commercial carbon fiber, manufactured by Zoltek (PX-35), labeled B. Zoltek PX-35 fibers were chosen as a baseline due to their prevalence in wind blade manufacturing [10]. Low-cost textile carbon fiber panels were manufactured using nine 160 K tows at a pull speed of 61 cm/min, while low-cost commercial fiber panels were manufactured using 22 50 K tows at a pull speed of 71 cm/min, as tabulated in Table 2. Panels were produced with a thickness measuring approximately 1.18 mm, a width measuring 62.5 mm, and were cut to lengths of approximately 1.2 m. A volume fraction of 55% was targeted for each panel. Additional panels, 2.97 mm by 99 mm by 1.2 m, were produced using the same manufacturing process and used for intentional defect testing.

### 2.3. Mechanical Testing Methods

Mechanical testing was performed using a hydraulic MTS axial test frame, equipped with a 100 kN capacity load cell. Data acquisition was achieved via MTS Multipurpose Elite software version 4.1.4.819. Strain gage data were recorded using National Instruments LabVIEW software version 11.0.

Large-span four-point flexure testing was conducted utilizing a custom test fixture having a support span of 287 mm, a load span of 127 mm, and a nodal radius of 12.7 mm for loading and support noses. A crosshead displacement of 25 mm was achieved at a crosshead displacement rate of 2 mm/min. Large-span flexure samples were shear cut to 51 cm length to accommodate the restrictions of the load frame while conserving as much of the original panel as possible.

To best understand the manufacturing quality, samples were tested as received, requiring minimal cutting of the original part. Due to restrictions of the load frame, the parts had to be cut shorter than as received, but the specimen overhang was quite large, approximately 110 mm on each side of the support span, removing interactions from possible damage accrued during cutting. The specimen width and support span exceeded the width of standard flexure fixtures; therefore, it was necessary to develop a custom fixture to accommodate the specimens.

**Figure 1:**
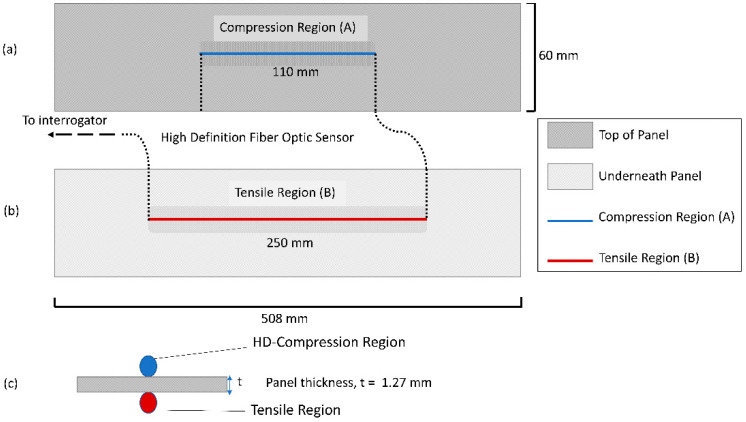
Schematic of high-definition fiber optic sensor (HD-FOS) mounted on the (**a**) tensile region and (**b**) compression results. (**c**) Cross-section of HD-FOS bonded to both facings of the pultruded carbon fiber-reinforced epoxy panels.

**Figure 2:**
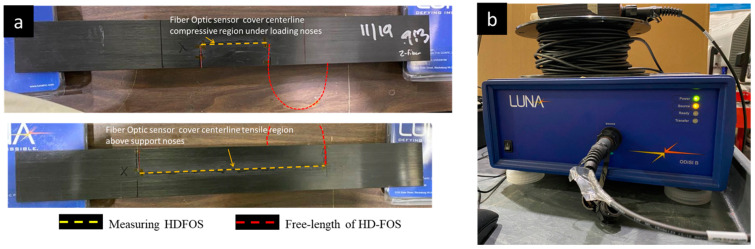
(**a**) HD-FOS bonded to the tensile and compression region of a unidirectional pultruded panel prior to flexural mechanical testing and (**b**) the LUNA ODiSI B interrogator unit.

**Figure 3:**
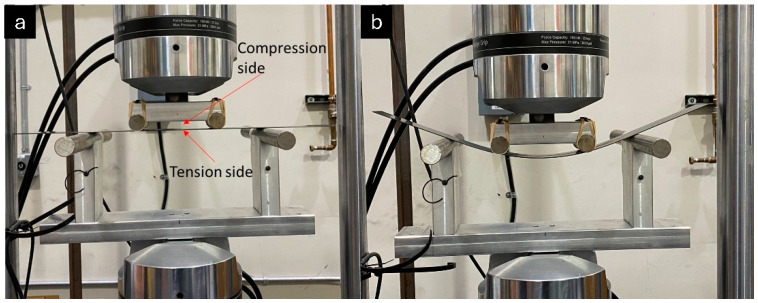
Custom large-span four-point flexural test configuration with HD-FOS mounted on pultruded beam specimen (**a**) prior to and (**b**) after mechanical loading.

**Figure 4:**
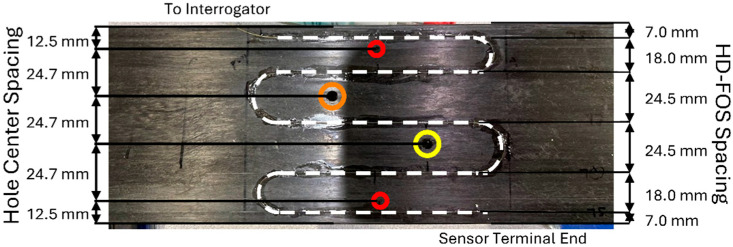
Pultruded carbon fiber-reinforced beam with drilled hole intentional defects equipped with HD-FOS mounted along the tensile face (dashed white line). HD-FOS was strategically placed to reveal the effects of hole size and their proximity to the sensor.

Initial large-span flexure tests utilized 350 ohm linear strain gages placed at the center of the loading span on the tensile and compressive faces to provide single point properties as a quick assessment of panel quality. However, manufacturing defects cannot be fully realized utilizing point properties, and spatial distribution of strain provides a thorough understanding of possible imperfections. The high spatial resolution strain distribution of the pultruded composite flexural samples (510 mm length × 60 mm width × 1.27 mm thickness) was measured using a 1 m long HD-FOS mounted along the centerline on both sides of the panel, as shown in the schematics in Figure 1 and Figure 2. The 155 μm outer diameter HD-FOS was bonded using a cyanoacrylate-based adhesive (M-Bond 200, Micro-Measurements, Rayleigh, NC, USA) along the samples on both sides of the tensile and compressive facings to monitor the longitudinal strain development of the panel subjected to flexural loading as pictured in Figure 3 [40]. Similarly, a second low-cost textile grade carbon fiber pultruding panel (510 mm length × 99.0 mm width × 2.97 mm thickness) with various diameter holes drilled within the maximum flexural stress region was equipped with a HD-FOS mounted to the tensile face of the specimen as shown in Figure 4 and tested on the same large-span flexure fixture.

**Figure 5:**
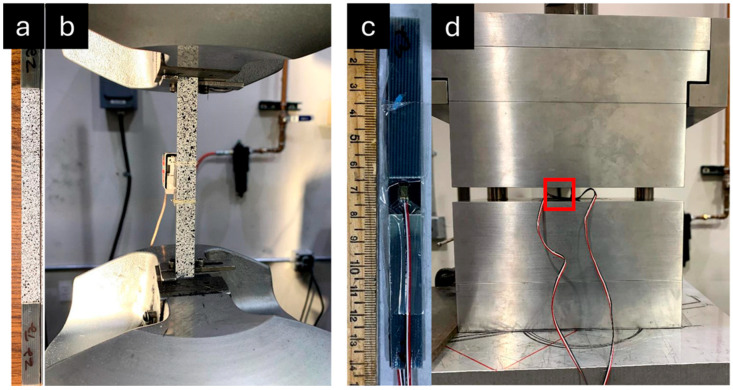
(**a**) Tensile specimens with speckle pattern for digital image correlation (DIC) strain measurement and (**b**) tensile specimen during testing with extensometer measuring strain along with DIC. (**c**) Compression specimens with strain gages attached to opposing faces and (**d**) IITRI compression fixture with specimens loaded (specimen highlighted by red box).

Upon the completion of large-span flexure testing, each of the non-defect pultruding panels was lightly sanded to remove the fiber optic sensor and adhesive residue. Coupon samples were extracted from the large-span flexure samples within the load span region for tension and compression mechanical property testing, as prescribed by ASTM D3039-17 and ASTM D3410-16, respectively. Samples and test fixtures for tension and compression testing are shown in Figure 5 [54,55].

One tensile specimen was extracted from each large-span flexure specimen with dimensions of 200 mm nominal length × 13 mm width × 1.27 mm thickness. G10 fiberglass tabs (50 mm length × 13 mm width × 1.6 mm thickness) were adhered to the specimens via a commercial cyanoacrylate adhesive (Loctite 401). Strain measurement was acquired via MTS extensometer (25 mm gage length), and spatial strain was monitored via digital image correlation (DIC) (Vic3D, Correlated Solutions, Irmo, SC, USA). A random speckle pattern was applied to the samples using flat white spray paint for a base with flat black spray paint speckles, as exhibited in Figure 5a.

One compression sample was extracted from large-span flexure specimens and tabbed with G10 fiberglass tabs (65 mm length × 13 mm width × 1.6 mm thickness), resulting in a 12.7 mm gage section. The 350 ohm linear strain gages were adhered to opposing faces of the specimens within the gage section via a cyanoacrylate-based adhesive (Loctite Plastics Bonding System), as shown in Figure 5c. Compression specimens were tested according to ASTM 3410-16, utilizing an IITRI compression test fixture (Model WF-II, Wyoming Test Fixtures, Salt Lake City, UT, USA), as displayed in Figure 5d [55].

### 2.4. Fiber Volume Fraction Analysis

The fiber volume fractions (FVFs) within the cross-section of the samples were examined and analyzed using a high-resolution digital optical microscope (Keyence Model VHX 7000, Itasca, IL, USA). Three specimens, approximately 25.4 mm × 20.8 mm × 1.27 mm (length by width by thickness) in size were extracted along the approximately 62.5 mm width of each pultruding panel type in preparation to visualize the complete area cross-section using optical microscopy. The specimens were mounted in a commercially available epoxy resin (Epoxicure 2, Lake Bluff, IL, USA) and cured at room temperate for a minimum of 8 h. The embedded samples were mounted into the sample holder where the edge of the samples was polished using a surface grinder (Buehler Metaserver 250). The edge of the samples was coarse grinded using grits 320 for 4 min, 600 for 6 min, and 800 for 8 min. The samples were then fine polished using 3 μm polycrystalline diamond (Buehler MetaDi Supreme)- and 0.05 μm alumina (Buehler Masterprep)-based suspensions. For each specimen, the sub-area cross-sections of the polished samples with an image size of approximately 4–5 mm length × 1.5–2 mm width were collected using the digital microscope. Each sub-area was digitally stitched using image processing software, ImageJ, coupled with image stitching software, MosiacJ, to observe the total cross-section of the panel. A custom developed algorithm generated by Barnett et al. was used to generate threshold images of the optical micrographs of sample cross-sections by employing an imaging processing technique to segment the pixels within each image into three unambiguous phases, namely (i) fiber, (ii) matrix, and (iii) void content [56]. The FVF and void content were then calculated based on the fraction area of the phases using the following relationships:

(5) $$FA=\frac{\sum Cross-section\;area\;of\;Fibers}{Total\;cross-section\;area\left(Fiber,Matrix,Void\right)},$$

where FA is the fiber area ratio and

(6) $$VC=\frac{\sum Cross-section\;area\;of\;Voids}{Total\;cross-section\;area\left(Fiber,Matrix,Void\right)},$$

where VC is the void content.

## 3. Results

### 3.1. Large-Span Flexure Test

**Figure 6:**
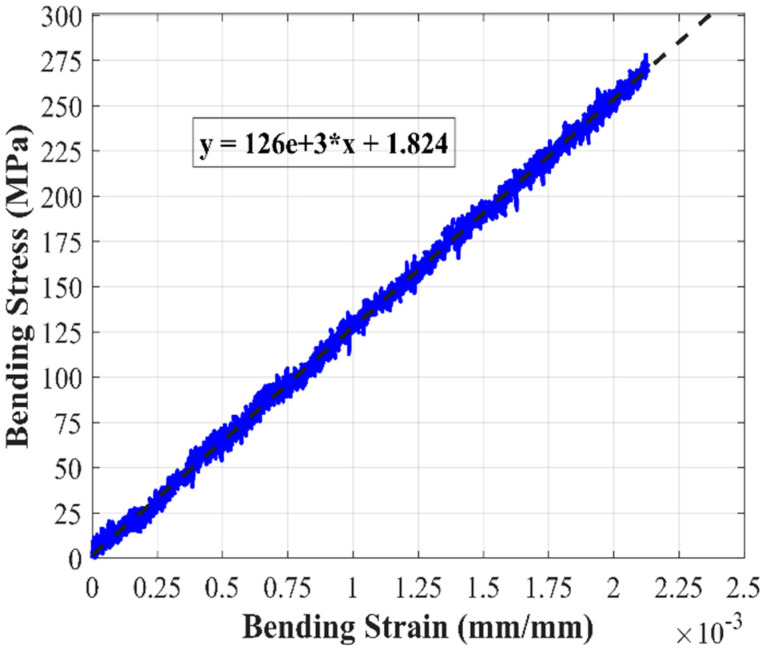
Example bending stress–strain curve with linear curve fitting for estimating bending modulus.

Bending moment was calculated using classic linear–elastic beam theory equation for four-point bending of simply supported beams, and bending modulus was taken as the linear best-fit correlation of the stress vs. average bending strain plot. An example of this is shown in Figure 6.

**Figure 7:**
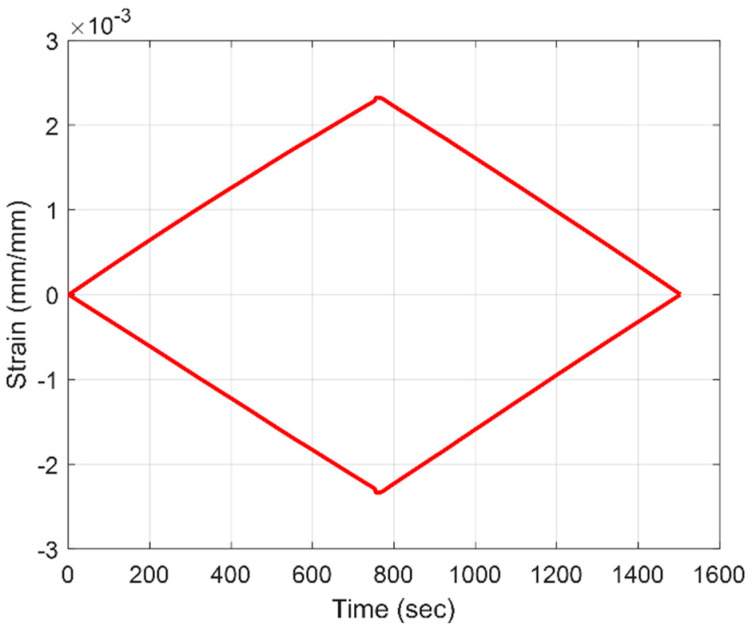
Compressive and tensile bending strains from strain gage data for a panel manufactured with commercial fiber B.

**Figure 8:**
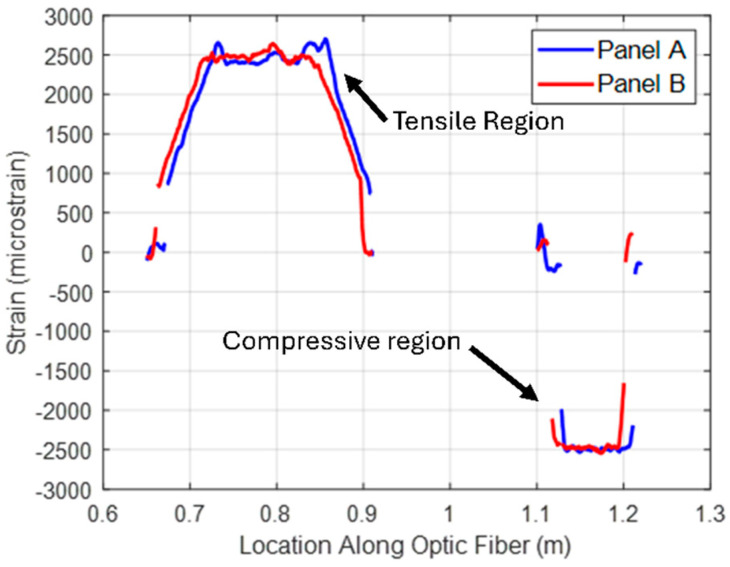
Comparison of strain distribution along HD-FOS observed at the maximum displacement of long span flexure testing for low-cost fiber Panel A and commercial fiber Panel B. Strain variations are more pronounced in the tensile region where the HD-FOS crosses the loading points for the bending fixture.

**Figure 9:**
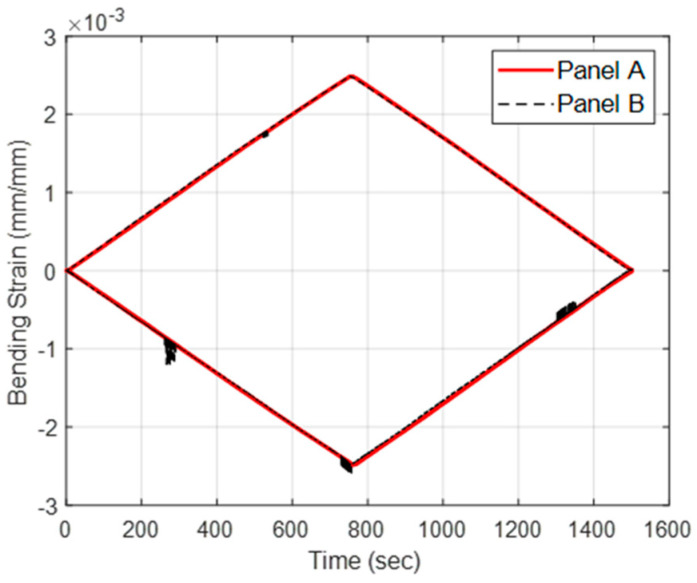
Average tensile and compressive strains of low-cost textile carbon fiber Panel A, and low-cost commercial carbon fiber Panel B during four-point flexure. The tensile and compressive strains are mirrored as expected.

Compressive strain and tensile strain were recorded during the flexural loading of large-span beams. During initial property assessment, strain gages were utilized to determine strain during bending, as shown in Figure 7. To capture the spatial distribution of strain along the sample length, an HD-FOS was utilized, as displayed in Figure 8. Strain variations were more pronounced in the tension region when compared to compression region near maximum deflection. However, these variations mainly occurred near the contact points of the loading nodes. Average compressive strain and tensile strain measurements from the fiber optic sensors were of identical magnitude for both panels, A (low-cost textile fiber) and B (low-cost commercial fiber), as shown in Figure 9, and were comparable to strain measurements from strain gage sensors in Figure 7. The large-span flexural modulus was 128 GPa for panel A and 127 GPa for panel B, an approximately 0.8% difference. The modulus for Panel B (commercial fiber-reinforced) aligns well with properties quoted for pultruding parts from the fiber manufacturer’s website. At 69% FVF, the quoted flexural modulus for these parts is 167 GPa. Using a linear proportional relationship, the flexural modulus reduces to 128 GPa when FVF is reduced to match that of the investigated pultruding panel at 53% FVF [57].

### 3.2. Extracted Coupon Test

**Figure 10:**
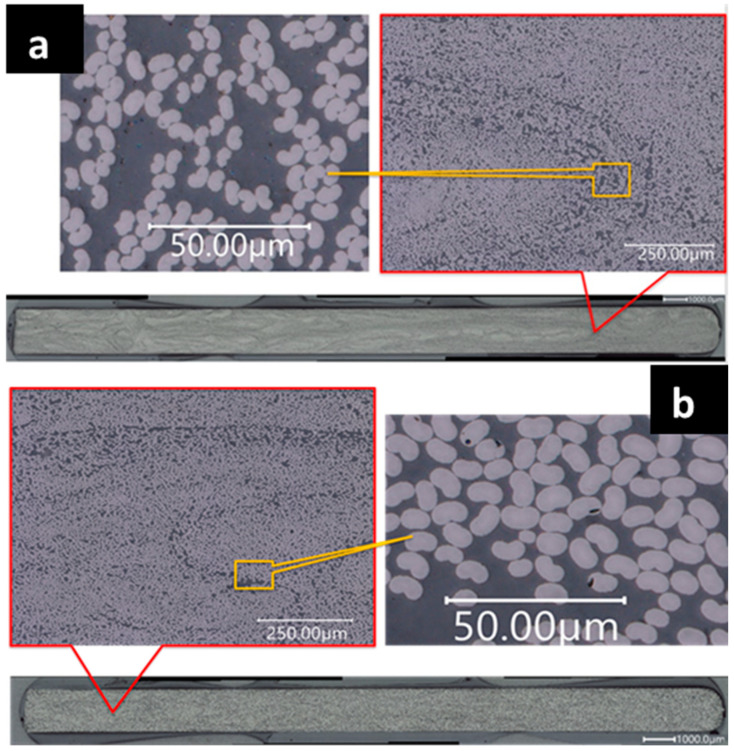
Cross-sectional scans using digital microscope to determine local fiber volume fractions for the (**a**) low-cost carbon fiber panel (Panel A) (**b**) commercial carbon fiber panel (Panel B).

Specimens were extracted from the large-span flexure beams and tested in tension and compression to obtain indicative mechanical properties imperative to the application of spar cap manufacturing. Table 3 summarizes the FVF, and the void content of example cross-sections of Panel A and Panel B is shown in Figure 10. Panel A FVF (54.33%) is slightly greater (2.39%) than the Panel B FVF (53.06%). Nearly zero percent void content was observed for both Panel A (0.03%) and Panel B (0.04%), indicating sufficient fiber wetout during the pultrusion manufacturing process. The mechanical properties obtained from these tests are presented in Table 4. The commercial fiber exhibited greater tensile modulus, while compressive strength was higher for the low-cost textile fiber. However, only one specimen for each test procedure was examined. To fully characterize these properties, further testing is required. ASTM D3039 and D3410 require at least five specimens to be tested for the results to be considered statistically significant [54,55]. Additionally, to ensure no bias exists across the width and along the length of the part, future specimens will be extracted from various locations across the width and along the length.

Buckling analysis obtained by comparing strain measurements from opposing faces of each specimen revealed significant buckling in both panels during compression loading. Panel A, containing low-cost textile carbon fiber reinforcement, exhibited 24% average buckling at failure, while Panel B, with low-cost commercial carbon fiber reinforcement, exhibited 16% average buckling at failure. Buckling is common when testing highly orthotropic thin pultruding parts due to the slenderness ratio of the fibers being significantly high, thus allowing the buckling of the individual fibers which progresses to the buckling of the part. ASTM 3410-16, recommends estimating maximum specimen gage length from Equation (7) to reduce buckling and allow uniform axial stress decay in compression by minimizing Poisson restraint effects produced by gripping [55,58]. Essentially, the specimen must be short enough to resist buckling, but long enough to allow stress decay from shear loaded compression. This equation is based upon the Euler buckling of a column with pinned ends where the effect length factor, k, is equal to 1.0. Utilizing this assumption, the Euler buckling equation reduces to Equation (7), where the constant 0.9069 approximates the ratios of constants from the Euler equation. In the equation gage length (l_g_) is based upon thickness (h) in mm, the longitudinal modulus of elasticity (E^C^) in MPa, ultimate compression strength (F^CU^) in MPa, and through-thickness shear modulus (G_xz_) in MPa. The ratio of longitudinal compressive strength to through-thickness shear modulus accounts for Poisson effects in buckling. For the panels produced in this work, a maximum gage length of 12.7 mm was calculated assuming G_xz_ of 4 GPa (based on ASTM suggestion), modulus of 128 GPa, and compression strength of 800 MPa. Due to the measured buckling being greater than 10% in both samples, further compression tests are needed to verify compression results using a gage length of 10 mm based upon the recommended minimum thickness of 1.2 mm from ASTM 3410-16.

(7) $$h\geq \frac{l_{g}}{0.9069\sqrt{\left(1-\frac{1.2F^{CU}}{G_{xz}}\right)\left(\frac{E^{C}}{F^{CU}}\right)}}$$

### 3.3. Large-Span Bending of Defect Panel

**Figure 11:**
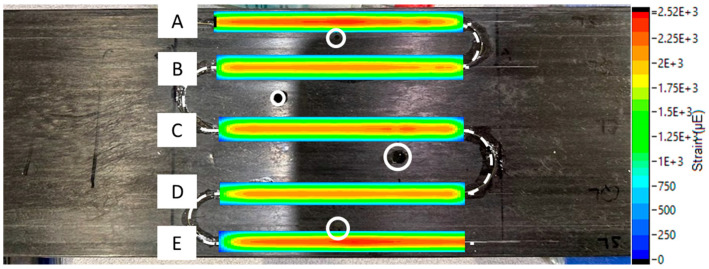
Strain concentrations near holes of original sizes at 10 mm displacement. Hole diameters from top to bottom were 2.49, 2.93, 1.32, and 1.93 mm, respectively. Letters A–E represent sensor regions.

**Figure 12:**
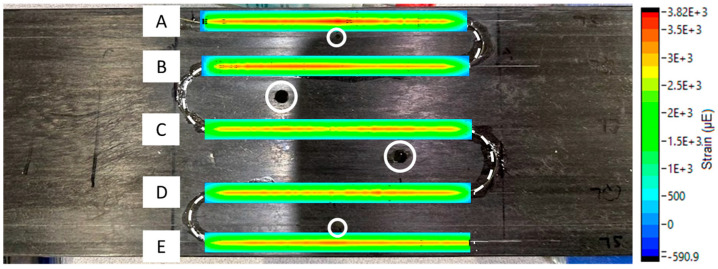
Strain concentrations near holes after hole size increased at 15 mm displacement. Hole diameters from top to bottom were 2.49, 5.08, 5.08, and 1.93 mm, respectively. Letters A–E represent sensor regions.

Strain concentrations varied based on hole size and proximity of the sensor to the defect. To better visualize strain variations, a commercial processing software, Integrated Data Acquisition and Analysis, iDAAS, version 1.0 (SIMAT Technologies, Manassas, VA, USA), was utilized. iDAAS was utilized both in situ and as a post-processing software, allowing the authors to create 2D contour plots that provided clear visual representations of the data. Figure 11 and Figure 12 reveal the importance of proximity to the defect and size of the defect to detect localized strain anomalies produced by the through-thickness defects. In Figure 11, contour plots of data revealed strain localizations near defects, especially near the edges of the panel (Regions A and E). Yet, data from sensor regions near the innermost defects (Regions B, C, and D) did not clearly emphasize the presence of defects. However, when the diameter of the innermost defects was increased, strain localizations were much clearer, as seen in Figure 12. These increases in strain concentration near defects highlight the reduction in the material’s ability to transfer load, which can lead to enhanced crack propagation; increased deflection, causing tower strikes; and premature failure.

**Figure 13:**
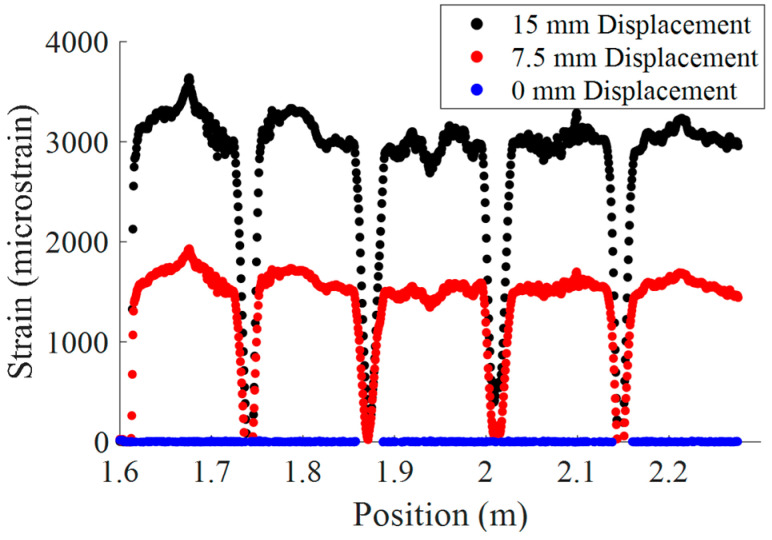
Scatter plot of HD-FOS strain during bending at 0 mm, 7.5 mm, and 15 mm displacement.

**Figure 14:**
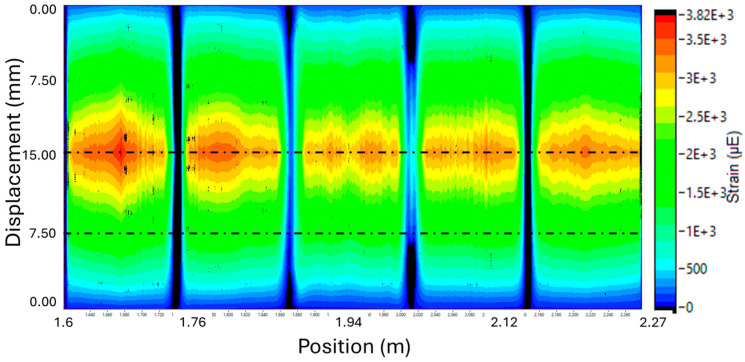
Two-dimensional contour plot of HD-FOS bending strain progression for a load/unload cycle with max displacement of 15 mm.

Figure 13 shows the strain distribution along the length of the fiber for 0 mm displacement, 7.5 mm displacement, and 15 mm displacement. As can be seen, peaks in strain occurred at positions where the HD-FOS was juxtaposed to the defects. The 2D contour map of the strain progression during a load/unload cycle, as shown in Figure 14, reveals these concentrations as they develop over time. The dashed lines were added to reveal the correlation to the scatter plot data from Figure 13. In Figure 14, strain concentrations are initially vague but develop quickly near the defect regions.

## 4. Discussion

Panel A manufactured with novel low-cost textile-grade fiber and Panel B manufactured with commercial low-cost fiber was tested for flexure, utilizing an HD-FOS to monitor the strain in situ. Comparable mechanical properties between Panel A and Panel B suggest the new textile-grade low-cost carbon fiber could be an exceptional candidate to replace commercial low-cost carbon fiber. However, a more complete regime of mechanical testing would be required to state this definitively. Future investigations into mechanical properties will involve multiple specimens taken from varied locations of the larger panel to remove local FVF effects. To meet requirements for ASTM D3039 and ASTM D3410, at least five specimens will be extracted for coupon tension and compression tests, and a two-tail t-test will be used to determine the statistical significance of the findings.

A pultruding panel made from the novel low-cost textile carbon fiber with intentional defects was subjected to flexure testing while monitoring strain via an HD-FOS. Strain localizations in the part with intentional defects were found to align well with approximations proposed by Konish and Whitney derived from the Lekhnittskiĭ method [59,60]. According to this method, stress concentrations around holes in laminated composites can be approximated as:

(8) $$\frac{{\bar{\sigma }}_{x}(0,y)}{{\bar{\sigma }}_{0}}≅1+\frac{1}{2}\rho^{-2}+\frac{3}{2}\rho^{-4}-\frac{k_{\sigma }-3}{2}(5\rho^{-6}-7\rho^{-8}),$$

where ${\bar{\sigma }}_{x}$ is the axial stress measured transversely from the hole edge, ${\bar{\sigma }}_{0}$ is the average applied far-field axial stress, ρ is the ratio of the distance from the hole edge to the hole radius and $k_{\sigma }$ is the ratio of the maximum circumfrential stress on the hole boudary to the applied far-field axial stress defined by Equation (9). In Equation (9), ${\bar{E}}_{x}$ is the average axial Young’s modulus, ${\bar{E}}_{y}$ is the average transverse Young’s modulus, ${\bar{\nu }}_{xy}$ is the average Poisson’s ratio, and ${\bar{G}}_{xy}$ is the average shear modulus. Since the applied stress was within the linear elastic region of the stress–strain curve, stress and strain are proportional based upon Young’s modulus.

(9) $$k_{\sigma }=\frac{{\bar{\sigma }}_{max}}{{\bar{\sigma }}_{0}}=1+\sqrt{2\left[\sqrt{\frac{{\bar{E}}_{x}}{{\bar{E}}_{y}}}-{\bar{\nu }}_{xy}\right]+\frac{{\bar{E}}_{x}}{{\bar{G}}_{xy}}}$$

**Figure 15:**
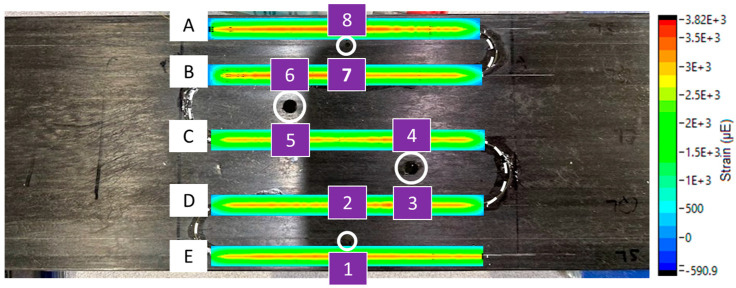
Labeling convention of regions laterally juxtaposed to defects investigated for local strain concentrations. Letters A–E represent sensor regions.

**Figure 16:**
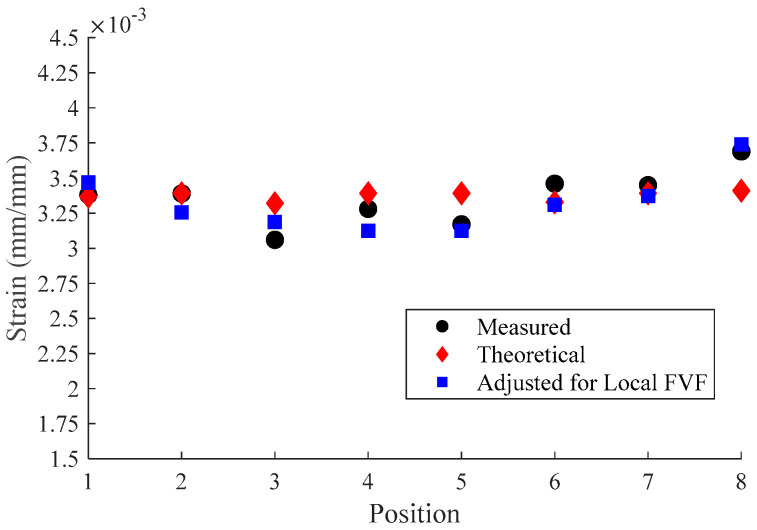
Strain localization results from measured experimental strain using fiber optic sensors, theoretical calculations, and adjusted theoretical calculations utilizing local FVF.

**Figure 17:**
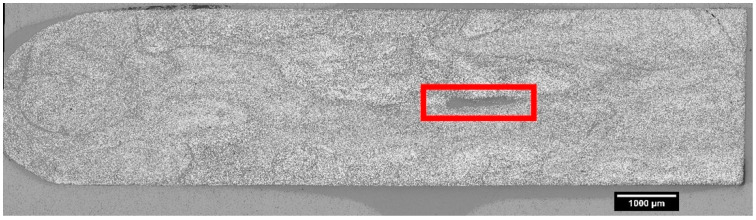
Optical microscopy of defect panel cross-section, revealing a resin-rich area.

Points along the fiber optic sensor laterally juxtaposed to the defects were labeled as shown in Figure 15. The strain calculated utilizing the average Young’s modulus was plotted for these positions in Figure 16. As can be seen from the figure, theoretical approximations align well with the experimental data apart from strain measured near the edge of the sample in Region A from Figure 12. The increased strain concentration in Region A near the outermost defect was likely due to decreased FVF at the location of the fiber optic sensor. Cross-sectional microscopy revealed a large pocket of resin near the sensor location, as shown in Figure 17. Although resin pockets are not common in pultruding parts, they can occur when fiber bundles are not tensioned properly during pultrusion, allowing the bundles to misalign and create voids where resin can be trapped. As this pultrusion line was still in development stages, mitigation strategies to address this issue, such as adjusting the fiber tension, are being developed. The local FVF was investigated to determine the effects of FVF on Young’s modulus. The FVF was measured to be 54.5% locally utilizing the method previously outlined. This is approximately 4.5% lower than the global FVF for the entire cross-section of the part. The rule of mixtures states that the composite modulus, E_C_, can be approximated as the weighted sum of the fiber modulus, E_F_, and the matrix modulus, E_M_, according to Equation (10) when voids are negligible. When E_C_ >> E_M_, as in carbon fiber P-FRC, the second term becomes negligible, allowing the composite modulus to be estimated based upon the fiber modulus and FVF only. We can adjust the local modulus from the global modulus using a ratio shown in Equation (11). Adjusting the strain by multiplying the calculated stress from Equation (8) by the local composite modulus saw better agreement with experimental values, as shown in Figure 16. Utilizing the approach of monitoring strain via HD-FOS coupled with an in situ visualization software like iDAAS presents a pathway to effectively assess the structural health of internal pultruded spar caps in wind blades.

(10) $$E_{C}=E_{F}*FVF+E_{M}*(1-FVF)$$

(11) $${\left[E_{C}\right]}_{local}={\left[\frac{E_{C}}{FVF}\right]}_{global}({FVF)}_{local}$$

## 5. Conclusions

The mechanical testing of two pultruded carbon-fiber beams was performed revealing comparable properties. Compressive strength comparison revealed a slight increase in the low-cost textile carbon fiber panel. The tensile modulus of the low-cost textile carbon fiber panel was about 10% lower than the commercial low-cost carbon fiber panel. However, spatial strain distribution revealed consistent strain measurement during four-point flexure for both material systems. The high-definition fiber optic sensing detected local strains throughout the part which would have been ambiguous if traditional strain sensing technology such as strain gages were used. The local strains detected by the fiber optic sensor were consistent throughout the loading zone, with spikes in the data occurring near the loading nodes for as-manufactured panels. Furthermore, HD-FOS was used to reveal localized strain concentrations near intentional defect regions during flexure testing. Some strain concentrations may have been enhanced due to local manufacturing defects where resin volume fraction was greater. Based on these results, with strategic placement of the fiber optic sensor via embedding or external adhesion, HD-FOS could be implemented to verify spatial mechanical properties post manufacturing and monitor those properties throughout the lifespan of the pultruded carbon fiber spar caps. This non-destructive testing approach is applicable for civil infrastructure applications, including the real-time monitoring of the structural behavior of fiber-reinforced composite-based bridges subjected to vehicular loadings, where the use of HD-FOS embedded within the bridge panel deck can measure the complex strain response spatially. Additional applicable case uses include but are not limited to automotive and aerospace applications evaluating the mechanical performance of post-manufactured parts consisting of various complex geometries in a range of environmental conditions.

The authors have presented a novel testing approach to evaluate both thick and wide samples that consider the combined effects of fiber interface, fiber bundle size and orientation, and resin volume fraction variations in typical pultruded carbon fiber composite materials using surface instrumented flexural testing with adequate length to thickness ratio as described in detail. The current approach of machining thick pultruded composites (thickness greater than 2 mm) using machined dog bone geometry both along thickness and width direction introduces unwanted changes at the nano-, micro-, and meso-scale features. The use of full thickness and large width parts, not possible in typical tensile or compression tests, using our proposed method, should pave the way for future characterization of high-strength and high-modulus pultruded composites, which are the future of the fiber-reinforced carbon composites space due to their low cost of manufacturing and exceptional properties.

## Figures and Tables

**Table 1:** Spectral shift unit values (GHz) conversion to strain units using calibration coefficients C_1_ and C_2_.

C_1_ (με/GHz)	C_2_ (με/GHz^2^)
−6.7000045109	−0.00005465390132

**Table 2:** Manufacturing specifications and fiber tow properties for pultruding parts.

Fiber Type	Linear Density (g/m)	Fibers/Tow	Tows/Panel	Pull Speed (cm/min)
A	8.67	160 K	9	61
B	3.77	50 K	22	71

**Table 3:** Fiber volume fraction (FVF) results for Panel A and Panel B.

Panel ID	Fiber Volume Fraction (%)	Void Content (%)
Panel A	54.33	0.03
Panel B	53.06	0.04

**Table 4:** Mechanical testing properties.

Test Type	Property	Panel A	Panel B
Large-Span Flexure	Modulus (GPa)	128	127
Tension	Modulus (GPa)	132	150
Compression	Strength (MPa)	833	798

## Data Availability

The raw data supporting the conclusions of this article will be made available by the authors on request. The data used in this study evaluates the performance of materials obtained from proprietary resources. Our aim is to protect proprietary information to prevent any potential unauthorized disclosure, supporting our commitment to providing transparent and reproducible research aligned with MDPI’s journal data availability policy.
